# Eye-size effects in the dot-probe task: Greater sclera exposure predicts delayed disengagement from fearful faces

**DOI:** 10.1371/journal.pone.0285839

**Published:** 2023-05-17

**Authors:** Jacob S. Aday, Lin Fang, Joshua M. Carlson

**Affiliations:** 1 Department of Psychiatry and Behavioral Sciences, University of California, San Francisco, California, United States of America; 2 Department of Psychological Science, Northern Michigan University, Marquette, Michigan, United States of America; University of Bologna: Universita di Bologna, ITALY

## Abstract

Fearful facial expressions are nonverbal and biologically salient signals of potential threat that automatically hold, capture, and direct observers’ attention. They are characterized by enlarged eye whites and dilated pupils, and fearful eyes alone are sufficient to capture attention. The morphological properties of the eye region, such as sclera exposure, are thought to play an important role in nonverbal communication. Specifically, increased sclera exposure associated with fearful expressions has been shown to moderate how observers’ shift their attention toward the direction of another’s gaze. Yet, the extent to which variability in sclera exposure possibly impacts the capture and hold of attention by fearful faces is untested. To address this, a sample of 249 adults completed a dot-probe task of selective attention with fearful and neutral faces. The results suggested that (1) fearful faces were prioritized over neutral faces (i.e., they captured and held attention), (2) greater sclera exposure at target locations facilitated reaction times, and (3) attention was held by greater sclera exposure of fearful faces at task irrelevant locations resulting in delayed disengagement. Collectively, the results indicate that fearful facial expressions and sclera exposure modulate spatial attention through independent and interactive mechanisms. Sclera exposure appears to be an important facilitator of nonverbal communication and perhaps represents an understudied variable in social cognition more broadly.

## Introduction

Fearful faces, characterized by enlarged eye-whites and dilated pupils [1], can serve as evolutionarily important signals that indicate the presence of a potential threat in one’s environment [2]. As such, they have been shown to selectively capture (i.e., facilitate orienting), hold (i.e., delay disengagement), and direct attention in order to facilitate adaptive responses [3,4]. The prototypical features of fearful faces (i.e., increased pupil size and eye-white area) serve to enhance sensory acquisition for both the expresser and observers [1,5]. A variety of state and trait variables have been shown to moderate the extent to which individuals attend to emotional faces [2,6,7], as have task-level variables such as stimulus-onset asynchrony [8–10]. Yet, little is known about how the morphology of facial expressions influences the capture and hold of observers’ attention.

A task that is commonly used to quantify the extent to which individuals attend to fearful faces, and emotional stimuli more broadly, is the dot-probe task [11–14]. The dot-probe task is a computerized cognitive test that begins with a central fixation point. After a period of fixation, two stimuli, one emotional and one neutral, are presented briefly (i.e., ~100–500 ms) on either side of the screen before being replaced with a target that participants must respond to as quickly as possible. Researchers often include three trial types: congruent (i.e., one emotional and one neutral face are presented; target appears behind the emotional face), incongruent (i.e., one emotional and one neutral face are presented; target appears behind the neutral face), and neutral (i.e., two neutral faces are presented). Faster responding on congruent relative to neutral trials is thought to reflect facilitated orienting, whereas slower responses on incongruent compared to neutral trials is indicative of delayed disengagement [4]. That is, if a participant’s attention is preferentially directed towards the threatening stimulus, then a subsequent target appearing in that location should be detected faster than if a neutral face had been there, and targets appearing away from fearful faces should take longer as the participant must disengage their attention from the location of the threat. By comparing congruent with incongruent trials, facilitated orienting and delayed disengagement can be collapsed into one general index of attentional bias toward threat [15,16].

Although much is unknown about which specific features of fearful faces underlie facilitated orienting and delayed disengagement in the dot-probe task, we do know that fearful eyes capture attention in and of themselves; that is, even when the rest of the face is cropped away, individuals still allocate more attentional resources to fearful compared to neutral eyes [17,18]. Moreover, fearful eyes elicit greater amygdala activity than neutral or happy eyes [19,20]. What could be driving this prioritization? Given that greater sclera exposure (i.e., white portion of the eyeballs) is related to increased amygdala activity and amygdala activation predicts prioritized gaze towards fearful eyes [21], differences in sclera exposure in the dot-probe task may contribute to the prioritization of fearful faces. Using a gaze cueing paradigm in which fearful and neutral faces gazed in competing directions, Carlson and Aday [3] found that increased sclera exposure in fearful eyes was related to facilitated responses in the cued location—indicating that sclera exposure is one factor underlying how fearful faces direct attention. Increased sclera exposure in fearful expressions was hypothesized to facilitate the attention of observers by making it more salient which direction the eyes were looking. It could be the case, then, that when faces are facing directly forward (such as in the dot-probe task), sclera exposure is also related to the capture and hold of attention by fearful faces. Although the role of sclera exposure in directing attention has been investigated [3], to-date, its possible role in the capture and hold of attention has not been tested.

The current study sought to identify if sclera exposure was related to the capture and hold of attention by fearful faces in the dot-probe task. We hypothesized that, on congruent trials, increased sclera exposure in the fearful face, relative to the neutral face, would be related to faster reaction times (RTs; i.e., sclera exposure is related to the initial capture of attention). We also hypothesized that, on incongruent trials, increased sclera exposure in the fearful face would be related to slower RTs (i.e., sclera exposure is related to the hold of attention at task irrelevant locations; Carlson and Aday [3]).

## Materials and methods

### Participants

249 adults (female = 184, right handed = 248) between 18 and 42 (*M* = 21.38, *SD* = 4.28) years of age with normal or corrected-to-normal vision participated in the study. These participants were individuals that completed a screening protocol as part of a clinical trial assessing the effects of attention bias modification on changes in brain structure [22]. Although not all participants included in this manuscript met inclusion criteria for the clinical trial, their screening data afforded a valuable opportunity to examine the possible role of sclera exposure in the dot-probe task with a large sample. The data utilized for analyses in this manuscript were collected during the screening session. Our sample size and design were based on the availability of this dataset. Participants received monetary compensation for their participation. Participants provided written informed consent before participation and the study was approved by the Northern Michigan University Institutional Review Board.

### Dot-probe task

The dot-probe task was programmed using E-Prime2 (Psychology Software Tools, Pittsburg, PA) and displayed on a 60 Hz 16” LCD computer monitor. Twenty fearful and neutral grayscale faces of 10 different actors (half female) were cropped to exclude extraneous features and used in the task. These stimuli were derived from the Karolinska Directed Emotional Faces (KDEF [23]) picturebank and a 3D facial database [24]. Database labels for fearful face stimuli included: 207F, 208F, 213F, 217F [19], AF14AFS, AF19AFS, AF22AFS, AM10AFS, AM22AFS, AM34AFS [23]; database labels for neutral face stimuli included: 207N, 213N, 217N [24], AF14NES, AF19NES, AF22NES, AM10NES, AM22NES, AM34NES (acronyms denote exact images used from each database) [23]. Ratings from a separate sample (*N* = 85) indicated that the fearful face stimuli were perceived as more negative (*M* = 3.83, *SD* = .30) than neutral faces (*M* = 4.45, *SD* = .52), *t* (18) = 3.23, *p* = .005) [25]. An independent samples t-test on image luminosity values indicated that fearful (*M* = 73.27, *SD* = 8.91) and neutral faces (*M* = 73.22, *SD* = 8.40) were balanced, *t*(18) = 0.01, *p* = .99. The standard deviation of the image intensity/luminosity is a measure of contrast (i.e., referred to as root-mean-square or RMS contrast [26]). These values were computed from the image histogram on Photoshop 22.5.0 and compared with an independent samples t-test. Again, there were no significant differences between fearful (*M* = 47.78, *SD* = 6.04) and neutral images (*M* = 47.42, *SD* = 7.06), *t*(18) = 0.12, *p* = .90.

**Fig 1:**
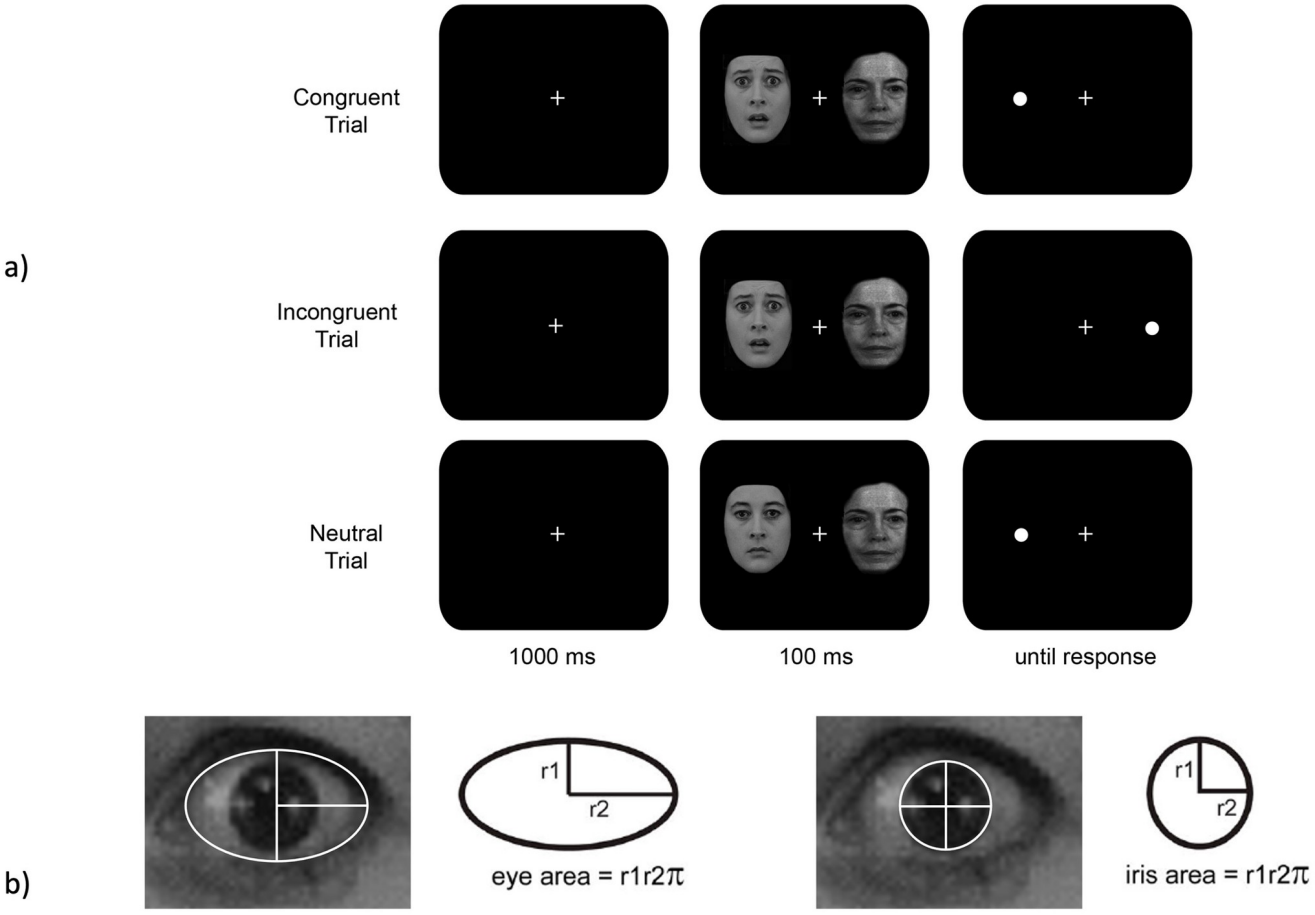
Task design and data analysis. (a) Congruent, incongruent, and neutral trials were included in the dot-probe task. (b) Measurements of total eye area and iris area were collected for each individual facial expression. Sclera exposure was quantified as the difference between these two measurements for each expression.

Participants were seated approximately 59 cm from the screen during the dot-probe task. As can be seen in Fig 1a, each trial started with a white fixation cue (+) in the center of a black screen for 1000 ms [27,28]. Two faces (5cm × 7cm) were simultaneously presented on the horizontal axis for 100 ms. Immediately after the faces disappeared, a target dot appeared at one of the two facial locations and remained on the screen until a response was made. Participants were instructed to focus on the central fixation cue throughout the trial and respond to the target dot as quickly as possible using a Chronos E-Prime response box. Participants indicated left-sided targets by pressing the first, leftmost button using their right index finger and indicated right-sided targets by pressing the second button using their right middle finger.

The task included congruent trials (dot on the same side as the fearful face), incongruent trials (dot on the same side as the neutral face), and baseline trials (two neutral faces). Faster responding on congruent trials relative to baseline is indicative of facilitated orienting, whereas faster responding on baseline compared to incongruent trials represents delayed disengagement [4]. The task consisted of five blocks with 450 trials in total. Each block contained 30 congruent, 30 incongruent, and 30 baseline randomly presented trials. At the end of each block, participants received feedback about their overall accuracy and RTs to encourage accurate rapid responses.

### Eye-size measures

As displayed in Fig 1b, the total eye area and iris area were calculated for the left and right eye individually and then averaged for each facial identity and expression [3]. Sclera area was calculated as the difference in total eye area–iris area. All eye-size measurements were highly correlated among one another (*r’s* = .82–.96); therefore, for brevity, we focused the present analyses on solely sclera measurements. Sclera area (ΔSA) difference scores were calculated as the average sclera area at correct locations − the average sclera area at incorrect locations for every face pairing across all trials of the experiment. Given that attention is often defined as the competition between two or more stimuli, ΔSA was calculated to reflect differences that would occur as a result of competition between sclera exposure across the two faces in the dot-probe task.

### Data preparation and analysis plan

Data were filtered to include only correct responses between 150 and 750 ms post-target onset to eliminate premature responses and lapses in attention; this common filtering practice attenuates noise while preserving the vast majority of responses (see: Carlson et al. [27]; Torrence et al. [28]; 96.46% of data was included). We analyzed the effects of ΔSA and Trial Type on RTs using a generalized linear mixed model (GLMM) with the identity link function and normal distribution for the RTs. Fixed effects contained main effects of ΔSA and Trial Type, ΔSA × congruency interaction, whereas the random effects contained the intercept for participants. To further analyze the expected interaction, separate GLMMs were conducted for congruent, incongruent, and baseline (i.e., neutral—neutral) conditions with the effect of ΔSA included as fixed effect and participant as random effect.

## Results

**Fig 2:**
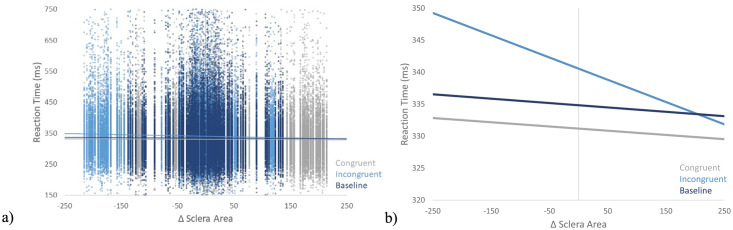
ΔSA and reaction time scatterplot. a) The relationship between ΔSA and reaction time is plotted for every individual trial. b) The relationship between ΔSA and reaction time is plotted by trial type. There was a significant relationship between ΔSA and reaction time on Incongruent, but not Congruent or Neutral trials.

A linear mixed model was used to assess the effects of ΔSA and Trial Type (Congruent, Incongruent, Neutral) on RTs. There was a main effect of Trial Type, *F*(2, 108,229) = 168.42, *p* < .001, *B*_Congruent-neutral_ = -3.52, 95%CI [-4.44, -2.60], *B*_Incongruent-neutral_ = 5.52, 95%CI [4.59, 6.45]. Simple contrasts indicate that participants responded faster on Congruent (*M* = 331.37, *SE* = 2.49) relative to Baseline (*M* = 334.89, *SE* = 2.49, *t* (108,229) = -7.47, *p* < .001, *d* = -0.47) trials, slower on Incongruent (*M* = 340.41, *SE* = 2.49) relative to Baseline (*t* (108,229) = 11.69, *p* < .001, *d* = 0.74) trials, and faster on Congruent relative to Incongruent trials (*t*(108,229) = -18.19, *p* < .001, *d* = 1.14). There was also a main effect of ΔSA, *F*(1, 108,229) = 34.71, *p* < .001, such that as ΔSA increased, RTs decreased. The Congruency × ΔSA interaction was significant, *F*(2, 108,229) = 24.03, *p* < .001, *B*_Congruent-neutral_ = -0.005, 95%CI [-0.018, 0.008], *B*_Incongruent-neutral_ = -0.04, 95%CI [-0.05, -0.02]. There was no effect of ΔSA on RTs during Congruent or Neutral trials (*p* > .05; Table 1). However, the effect of ΔSA on RTs was significant during Incongruent trials, such that increased sclera exposure of fearful faces at the incorrect/incongruent location was related to increased RTs (*p* < .001; Fig 2).

**Table 1:** Statistics for the Trial Type × ΔSA interaction in a linear mixed model on reaction time.

Congruency	Estimate (*b*)	95% CI	Test Statistic (*t*)	p-value
Congruent	-0.006	[-0.013, .001]	-1.66	.097
Incongruent	-0.037	[-.044. -.030]	-10.09	< .001
Neutral	-0.001	[-.012, .009]	-0.223	.823

## Discussion

In this study, we sought to determine the extent to which sclera exposure may influence the capture and hold of attention by fearful faces in the dot-probe task. ΔSA was calculated as the average sclera area of faces preceding correct target locations − the average sclera area of faces preceding incorrect target locations for each trial in the experiment. Consistent with a large body of literature [2,4,12,13] we found that fearful faces captured and held attention in the dot-probe. In addition to this confirmatory finding, the study produced two novel effects. First, across all trials, greater ΔSA was associated with faster RTs suggesting that increased sclera exposure of faces preceding the target location facilitated RTs. Second, there was an interaction between Congruency and ΔSA, such that larger sclera exposure of fearful faces at incongruent locations led to slower RTs. This finding suggests that increased sclera exposure in fearful faces was related to the hold of attention by fearful faces at task irrelevant locations. Thus, fearful faces were found to modulate attention in the dot-probe task and this modulation of attention was (at least in part) linked to sclera exposure.

Across all trials in the experiment, the relative difference in sclera exposure between faces preceding the target location and those at the opposite location influenced RTs. Specifically, greater relative sclera exposure at the correctly cued location facilitated RTs suggesting that sclera exposure is a salient feature of faces that impacts observers’ attention. This finding is similar to a recent report indicating that larger pupil sizes facilitate attentional bias to the eye region of faces [29]. Thus, it appears that both pupil size and sclera exposure modulate attention, which may not be surprising given the strong correlation between these measures observed in the present report. It should be noted that these effects seem to reflect a facilitation of RTs independent of facial expression. It should also be noted that although fearful faces are characterized by enlarged eye whites, not all fearful face identities had greater sclera exposure than neutral face identities. This general influence of sclera exposure on attention highlights the salience of eyes in nonverbal social communication. Indeed, the eye region signals a variety of emotional signals [30–33] and the location of others’ attentional focus [34]. The whiteness of the sclera and the degree to which it is exposed are unique morphological features in humans (relative to other primates) that are thought to allow for greater eye movement and nonverbal communication [35]. Given the importance of sclera exposure in multiple aspects of nonverbal communication, the broad modulation of attention by enlarged eye whites may be an adaptive means of detecting important social signals from expressers.

We also found that greater sclera exposure in fearful faces at locations opposite the target were proportionally linked to delayed target detection. This suggests that fearful faces with enlarged sclera held attention at this task irrelevant location longer than fearful faces with minimal sclera exposure. Previous research has parsed attentional bias in the dot-probe task into the initial orienting or capture of attention as well as the hold of attention (i.e., delayed disengagement) [15,16]. Research suggests that when attention is captured by fearful faces in the dot-probe task, it can be attributed to both the initial grab and subsequent hold of attention [4,23]. Other research using non-facial stimuli in high-anxious individuals suggests that attention effects in the dot-probe task are primarily attributed to the hold (or delayed disengagement) of attention by threatening stimuli [36,37]. Here, we provide novel evidence that delayed disengagement from fearful faces is specifically linked (at least in part) to sclera exposure. On the other hand, the initial capture of attention by fearful faces was not associated with sclera exposure and therefore appears to be mediated by some other mechanism. Perhaps the initial capture of attention is driven more by the classification of the stimulus/face as threat-related or socially significant rather than the physical attributes of the stimulus as we had predicted.

Indeed, in addition to the influence of sclera exposure on attentional bias, we also found a main effect of congruency suggesting that fearful faces capture and hold attention relative to neutral faces. This finding is consistent with decades of research suggesting that fearful faces and other threatening or emotional stimuli modulate attention [2,4,38–42]. Here, we demonstrate that fearful faces elicit a robust capture of attention even when other factors such as sclera exposure are included in the statistical model. In addition, we provide evidence that aspects of attention to fearful faces are linked to sclera exposure (i.e., the hold of attention) whereas other aspects (i.e., the initial orienting of attention) appear to be independent of sclera exposure and likely linked to some other characteristic/attribute of the fearful face.

### Limitations and future directions

There are limitations to keep in mind when interpreting the current findings as well as important areas of future research to consider. First, it should be noted the effect size of sclera exposure was small, but statistically significant and in the hypothesized direction, in this instance. Additionally, as this study was correlational in nature, future research that experimentally manipulates sclera size will be needed to ensure these findings are not due to other facial features which may covary with changes in sclera size. It may also be useful to present neutral and emotional eye-whites in isolation and compare against a control condition with a reverse contrast (e.g., Whalen et al. [20]). Lastly, future research will be needed to determine if the effects observed here for fearful faces generalize to other emotional expressions.

### Conclusion

The morphological characteristics of emotional facial expressions play an important role in nonverbal communication and social cognition. In particular, observers rely on the eye region for critical social cues, but little is known about specific features of the eyes that influence observers’ attention. Here, with a large sample size, we found that greater sclera exposure facilitated reaction time and that increased sclera exposure in fearful expressions specifically held attention. Thus, the current results provide novel evidence that sclera exposure and fearful facial expressions modulate attention through both independent and interactive mechanisms.

## Supporting information

S1 File (SAV)
